# Clinical, Diagnostic, and Imaging Findings in Three Juvenile Dogs With Paraspinal Hyperesthesia or Myelopathy as a Consequence of Hemophilia A: A Case Report

**DOI:** 10.3389/fvets.2022.871029

**Published:** 2022-04-15

**Authors:** Kayla M. Fowler, Timothy A. Bolton, John H. Rossmeisl, Avril U. Arendse, Karen M. Vernau, Ronald H. L. Li, Rell L. Parker

**Affiliations:** ^1^Department of Small Animal Clinical Sciences, Virginia-Maryland College of Veterinary Medicine, Virginia Tech, Blacksburg, VA, United States; ^2^Comprehensive Cancer Center and Brain Tumor Center of Excellence, Wake Forest School of Medicine, Winston-Salem, NC, United States; ^3^Department of Surgical and Radiological Sciences, University of California, Davis, Davis, CA, United States

**Keywords:** hemophilia, myelopathy, paraspinal hyperesthesia, magnetic resonance imaging (MRI), hemorrhage

## Abstract

Three juvenile dogs presented with an acute onset of paraspinal hyperesthesia and/or neurologic deficits. These dogs underwent anesthesia for MRI and additional diagnostics. The thoracolumbar MRI in Dog 1 revealed an accumulation of T2-weighted (T2W) hyperintense, T1-weighted (T1W) iso- to hyperintense, contrast enhancing extradural material. The differential diagnoses were meningitis with secondary hemorrhage or empyema or late subacute hemorrhage. The initial cervical MRI in Dog 2 revealed T1W meningeal contrast enhancement suspected to be secondary to meningitis. A repeat MRI following neurologic decline after CSF sampling revealed a large area of T2W and T1W hyperintensity between fascial planes of the cervical musculature as well as T2W iso- to hyperintense and T1W iso- to hypointense extradural material at the level of C1 consistent with hemorrhage. The cervical MRI in Dog 3 revealed T2W hyperintense and T1W iso- to hypointense extradural compressive material consistent with hemorrhage. Dogs 1 and 2 underwent CSF sampling and developed complications, including subcutaneous hematoma and vertebral canal hemorrhage. Dog 3 underwent surgical decompression, which revealed a compressive extradural hematoma. In each case, a hemophilia panel including factor VIII concentration confirmed the diagnosis of hemophilia A. Dog 1 had a resolution of clinical signs for ~5 months before being euthanized from gastrointestinal hemorrhage. Dog 2 was euthanized due to neurologic decompensation following CSF sampling. Dog 3 did well for 2 weeks after surgery but was then lost to follow-up. This case series provides information on clinical signs, MRI findings, and outcome in 3 juvenile dogs with hemophilia A that developed neurologic deficits or paraspinal hyperesthesia secondary to spontaneous or iatrogenic vertebral canal hemorrhage. Hemophilia A should be considered as a differential in any young dog presenting with an acute onset of hyperesthesia with or without neurologic deficits. This diagnosis should be prioritized in young male dogs that have other evidence of hemorrhage on physical exam.

## Introduction

Spontaneous hemorrhage in the vertebral canal secondary to hemophilia A is infrequently reported. Three cases of German Shepherd Dogs with hematorrhacis secondary to hemophilia A have been described (1). One of those cases had myelographic findings of an extradural lesion location, though necropsy confirmed subdural and epidural hemorrhage. A case report described a 1-year-old Labrador Retriever-Terrier mix that presented for cervical pain and was diagnosed with vertebral canal hemorrhage using CT (2). This dog initially recovered and was diagnosed with Hemophilia A; however, the dog was reported to experience spontaneous gastrointestinal and hemarthroses 5 months later. A 7-week-old chow chow-keeshond mix that presented for acute paraplegia with absent nociception and episcleral hemorrhage was confirmed to have hemophilia A by clotting factor analysis. Imaging was not performed, but necropsy revealed extradural and subdural hemorrhage (3). Finally, a dog diagnosed with hemophilia A was euthanized after developing a myelopathy, but the underlying cause of the myelopathy was not confirmed (4).

Magnetic resonance imaging characteristics of hemorrhage into the vertebral canal have been reported previously in several case studies (5–9). Vertebral canal hemorrhage may be secondary to coagulopathy, neoplasia, trauma, intervertebral disc herniation, acute non-compressive nucleus pulposus extrusion, inflammatory diseases, iatrogenic, traumatic, or may be idiopathic (5, 7–14). The progression of hemoglobin degradation and therefore the imaging findings may proceed differently than in the brain due to variability in the local environment, but this has not been established in the dog (5, 15, 16). T2^*^ weighted gradient echo imaging (T2^*^) may be useful to identify a hemorrhagic lesion (6). In a series of cases with spontaneous vertebral canal hematomas, the MRI findings were variable with the lesion being T2W hyperintense in 5/6 cases and T1W isointense in 4/6 cases. Only one case exhibited gadolinium contrast enhancement (5). In the case that had a T2^*^ sequence performed, susceptibility artifacts were identified. This study did not find a strong correlation between lesion age and imaging findings. This case report describes the clinical, diagnostic, and MRI findings of three dogs with confirmed Hemophilia A.

## Case Presentations and Outcomes

### Case 1

A 4-month-old 4.0 kg sexually intact male Dachshund (Dog 1) was evaluated for an acute onset of paraspinal hyperesthesia. At initial evaluation by the referring veterinarian, the dog was suspected to have abdominal pain and had normal abdominal radiographs. The dog was treated with a tapering course of prednisone but worsened when the prednisone was discontinued, and was referred for further diagnostics approximately 1 month later. On presentation, neurologic examination revealed ambulatory paraparesis with a mild to moderate proprioceptive ataxia, mildly delayed proprioception placement and hopping in the pelvic limbs, and a low head carriage with cervical and thoracolumbar hyperesthesia. The neuroanatomic localization was a T3–L3 myelopathy with concurrent cervical hyperesthesia or multifocal with evidence of a C1–C5 and T3–L3 myelopathies. A cervical myelopathy could not be excluded. The remainder of physical examination findings were unremarkable.

A CBC revealed a leukocytosis (22,440 WBCs/ul; reference range, 5,050–16,760 WBCs/ul), mature neutrophilia (15,932 neutrophils/ul; reference range, 2,455–9,170 neutrophils/ul), and lymphocytosis (5,386 lymphocytes/ul; reference range, 913–3,281 lymphocytes/ul). The CBC also revealed a mild normocytic and normochromic anemia (Hematocrit 36.5%; reference range, 37.3–61.7%). Serum biochemical analysis revealed a mildly increased alkaline phosphatase (149 U/L; reference range, 8–70 U/L), hyperphosphatemia (6.9 mg/dl; reference range, 1.9–4.4 mg/dl), hypercalcemia (11.0 mg/dl; reference range, 9.4–10.7 mg/dl), mildly decreased creatinine (0.28 mg/dl; reference range, 0.7–1.3 mg/dl), mildly increased creatine kinase (218 U/L; reference range, 32–193 U/L), and mild hypochloremia (109 mEq/L; reference range, 110–119 mEq/L). All other laboratory values were within reference ranges.

**Figure 1:**
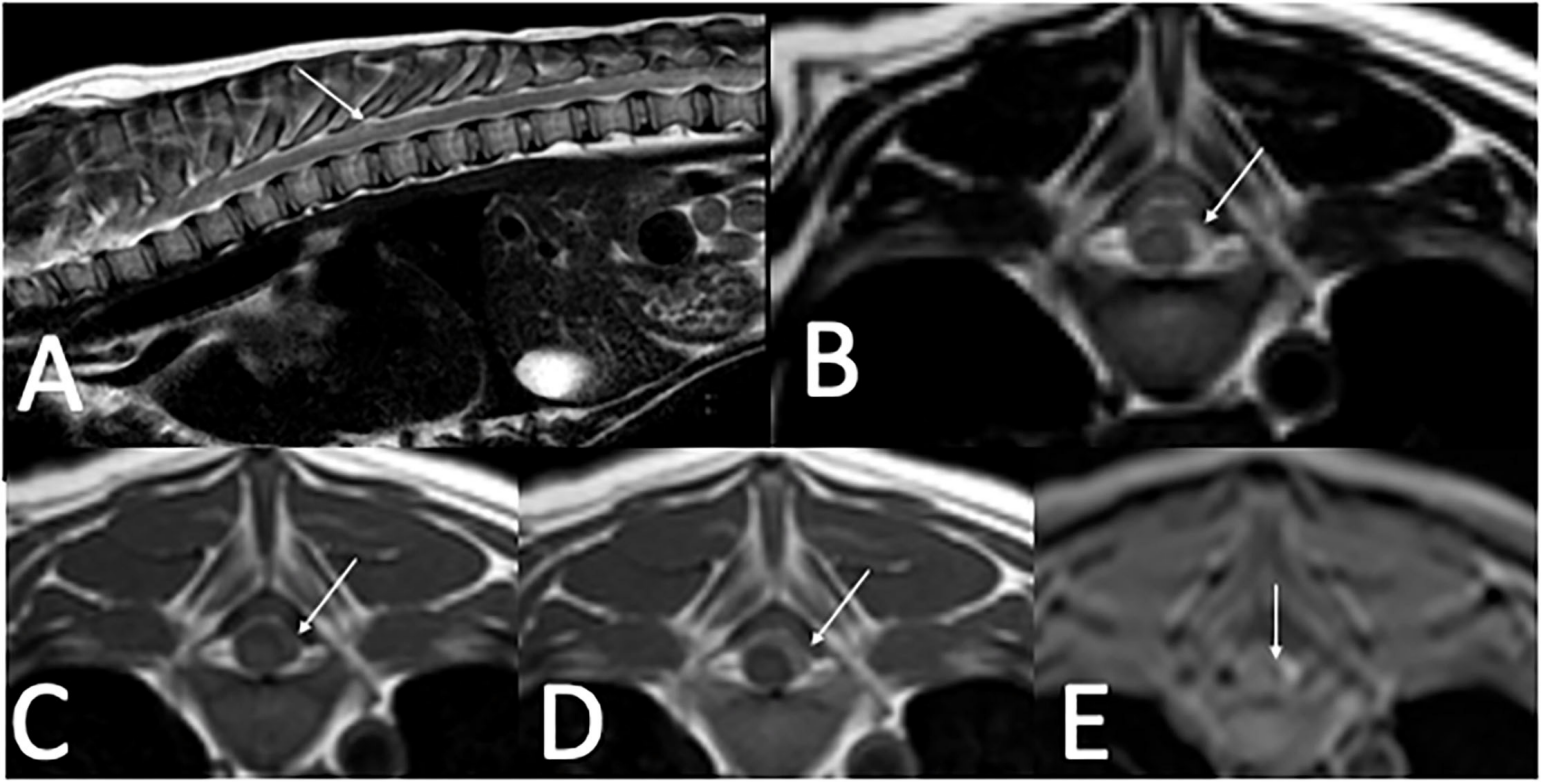
Thoracolumbar MRI in a 4-month-old 4.0 kg sexually intact male dachshund with an acute onset of diffuse paraspinal pain and T3–L3 myelopathy (case one). **(A)**—T2W sagittal image of the thoracolumbar spine showing heterogenous extradural T2 hyperintensity located between T3–T8 (arrow). **(B)**—T2W transverse image at the level of T8 with T2 heterogenously hyperintense extradural material in the left dorsolateral vertebral canal (arrow). **(C)**—T1W pre-contrast image at the level of T8 with T1 iso- to hyperintense extradural material in the left dorsolateral vertebral canal (arrow). **(D)**—T1W post-contrast image at the level of T8 with mild homogenous contrast enhancement of the extradural material in the left dorsolateral vertebral canal (arrow). **(E)**—T2* image at the level of T8 with hyperintense extradural material in the left dorsolateral vertebral canal and small T2* signal void, consistent with a susceptibility artifact medial to the hyperintense material (arrow).

Cervical radiographs were normal, ruling out atlantoaxial malformation or instability. A thoracolumbar and cervical MRI performed 3 days after referral revealed T2-weighted (T2W) and STIR heterogeneously hyperintense, T1-weighted (T1W) iso- to hyperintense, T2^*^ hyperintense, homogenously contrast enhancing extradural material that extended from T3 to cranial T8 in the left lateral aspect of the vertebral canal. On T2^*^, there was a small extradural signal void consistent with a susceptibility artifact at the level of T8. This material resulted in mild spinal cord compression. MRI also revealed multifocal T2W hypointense *in situ* intervertebral discs (Figure 1). Images were acquired with a 1.5T MRI^1^. Differentials following MRI included meningitis with secondary empyema or hemorrhage and neoplasia.

Lumbar CSF sampling was attempted, and needle placement was confirmed with fluoroscopy, but an adequate sample volume could not be obtained. Cerebellomedullary cisternal CSF analysis was performed and revealed increased leukocytes (148 WBC/ul; reference range, 0–3 WBC/ul), increased erythrocytes (221 RBC/ul; reference range, 0–3 RBC/ul), and normal protein (20.9 mg/dl; reference range, ≤ 25 mg/dl). Cytology revealed a moderate mixed inflammatory population, comprising 48% quiescent to mildly activated large mononuclear cells that occasionally contained phagocytized erythrocytes or hematoidin crystals, 39% nondegenerate neutrophils, and 13% small to intermediate lymphocytes. A small subcutaneous hematoma was noted at the site of CSF collection. Treatment for suspected meningitis was initiated, which included prednisone 0.65 mg/kg PO Q12hr, clindamycin 12.5 mg/kg PO Q 12 h, and gabapentin 25 mg/kg PO Q12hr.

Following recovery from anesthesia, the dog developed severe subcutaneous hemorrhage in the left pelvic limb. Active hemorrhage was visible from the site of the recently removed arterial catheter. Subcutaneous hemorrhage continued despite 24 h of compression bandages and ice packing. Clotting times revealed a normal prothrombin time (PT) (7.9 s; reference range, 7.3–8.8 s) and prolonged activated partial thromboplastin time (aPTT) (19.5 s; reference range, 9.9–12.1 s). Blood typing was DEA 1.1 positive. Serial packed cell volume measurements over the ensuing 12 h revealed a worsening anemia, indicating ongoing hemorrhage (35% when hemorrhage was first observed, 19% 6 h later, and 14% 12 h later). A DEA 1.1 negative packed red blood cell transfusion was administered over 4 h given the dog's clinical signs suggestive of shock (tachycardia with a heart rate of 160 bpm, hypertensive with a systolic blood pressure of 150 mmHg measured by Doppler, and development of a grade II/VI left apical systolic heart murmur). Differentials for the spontaneous hemorrhage included arterial laceration or coagulopathy, including Von Willebrand's disease or Hemophilia. Cryoprecipitate at a dose of 1 unit/dog (70 ml unit) was administered once IV over 5 h. Desmopressin was administered as a single 0.75 μg/kg SQ dose. Spontaneous hemorrhage had resolved upon discontinuation of the cryoprecipitate infusion and the dog was comfortable with no obvious hyperesthesia 2 days later. A hemophilia panel was not submitted at this time due to the patient having received a blood transfusion and cryoprecipitate, which may have affected the results. This dog's peripheral veins were also compromised from repeated sampling, and blood could only be obtained with a jugular blood draw at the time of discharge. It was judged to not be in the dog's best interest to take a jugular sample at that time.

The dog was discharged with gabapentin 25 mg/kg PO Q12hr, prednisone 0.65 mg/kg PO Q12hr, and clindamycin 12.5 mg/kg PO Q12hr, with instructions to return through the Internal Medicine service for additional coagulopathy diagnostics. At the recheck appointment 3 weeks later, physical exam revealed a 4–5 cm ecchymosis on the right ventral abdomen adjacent to the prepuce. IgA antibody testing was normal (47 mg/dl; reference range, 35–270 mg/dl). A hemophilia panel revealed a factor VIII deficiency (5%; reference range; 50–200) consistent with a diagnosis of hemophilia A. Infectious disease testing performed at the time of initial evaluation was negative for Neospora, Toxoplasma, Blastomyces, Lyme, Rocky Mountain Spotted Fever, and Ehrlichia. Gabapentin and clindamycin were both discontinued. Prednisone was discontinued following a 1-week taper. This dog did well for another 5 months before developing gastrointestinal hemorrhage suspected to be secondary to a gastrointestinal obstruction. The owners elected euthanasia without a necropsy.

### Case 2

An 11-month-old 8.3 kg sexually intact male Dachshund (Dog 2) was evaluated for an acute onset of cervical pain. Physical examination revealed scleral hemorrhage of the left eye but was otherwise unremarkable. Neurologic examination revealed severe cervical paraspinal hyperesthesia with no neurologic deficits. The primary problem was cervical paraspinal hyperesthesia. A CBC revealed a leukocytosis (16,950 WBC/ul; reference range, 5,000–15,550 WBC/ul), mature neutrophilia (10,679 neutrophils/ul; reference range, 2,455–9,170 neutrophils/ul), lymphocytosis (3,729 lymphocytes/ul; reference range, 913–3,281 lymphocytes/ul), monocytosis (1,187 monocytes/ul; reference range, 79–1,160 monocytes/ul), and thrombocytosis (427,000 thrombocytes/ul; reference range, 150,000–393,000 thrombocytes/ul). Serum biochemical analysis revealed hypercalcemia (11.4 mg/dl; reference range, 9.4–10.7 mg/dl) and hyperphosphatemia (5.9 mg/dl; reference range, 1.9–4.4 mg/dl).

**Figure 2:**
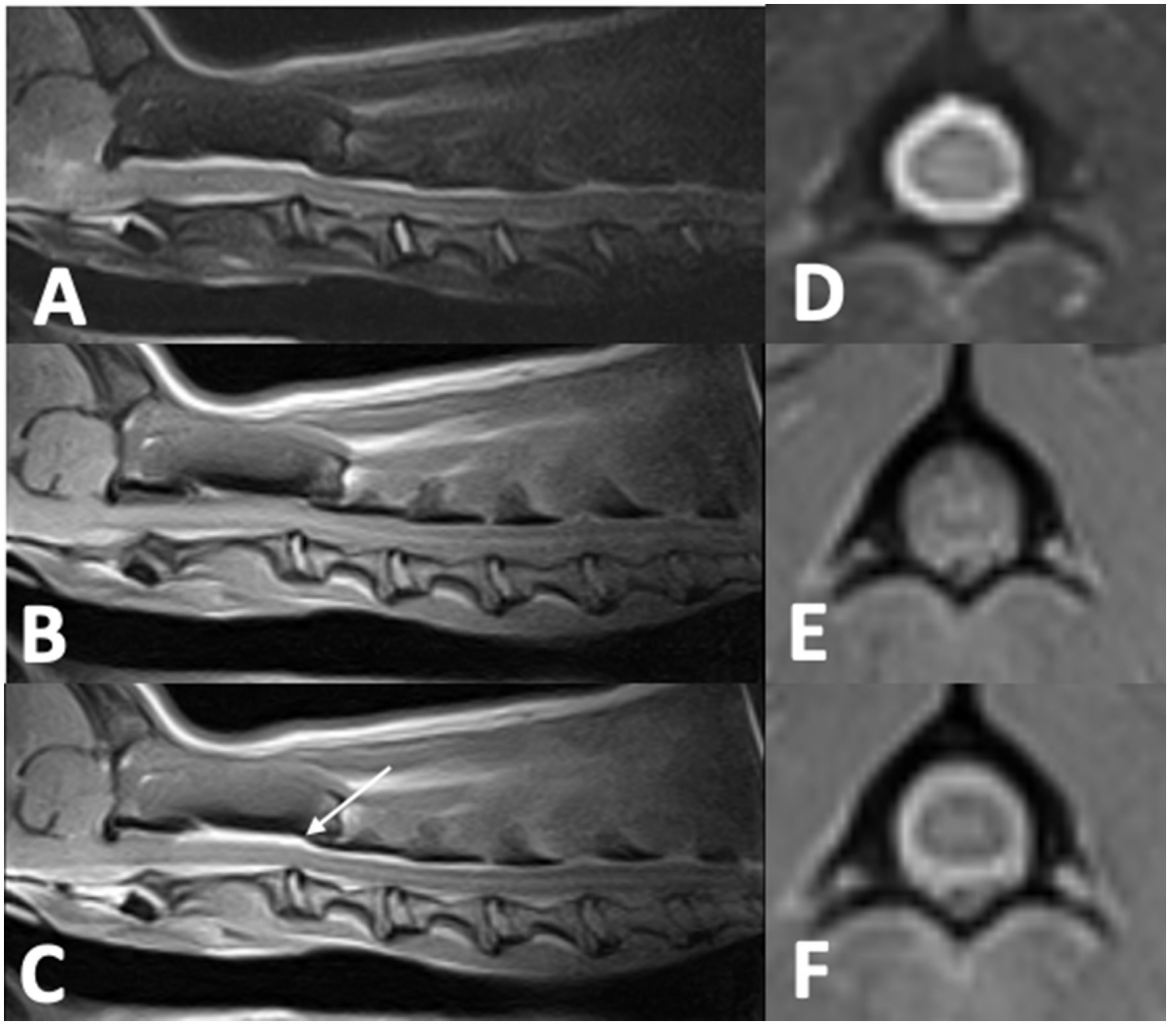
Cervical MRI in an 11-month-old 8.3 kg sexually intact male dachshund with an acute onset of cervical pain (case two). **(A)**—T2W sagittal image of the cervical spine that is unremarkable. **(B)**—T1W sagittal image of the cervical spine that is unremarkable. **(C)**—T1W post-contrast sagittal image of the cervical spine with dorsal and ventral meningeal contrast enhancement from C1–C4 (arrow). **(D)**—T2W transverse image at the level of C1 that is unremarkable. **(E)**—T1W transverse image at the level of C1 that is unremarkable. **(F)**—T1W post-contrast image at the level of C1 showing circumferential meningeal contrast enhancement.

**Figure 3:**
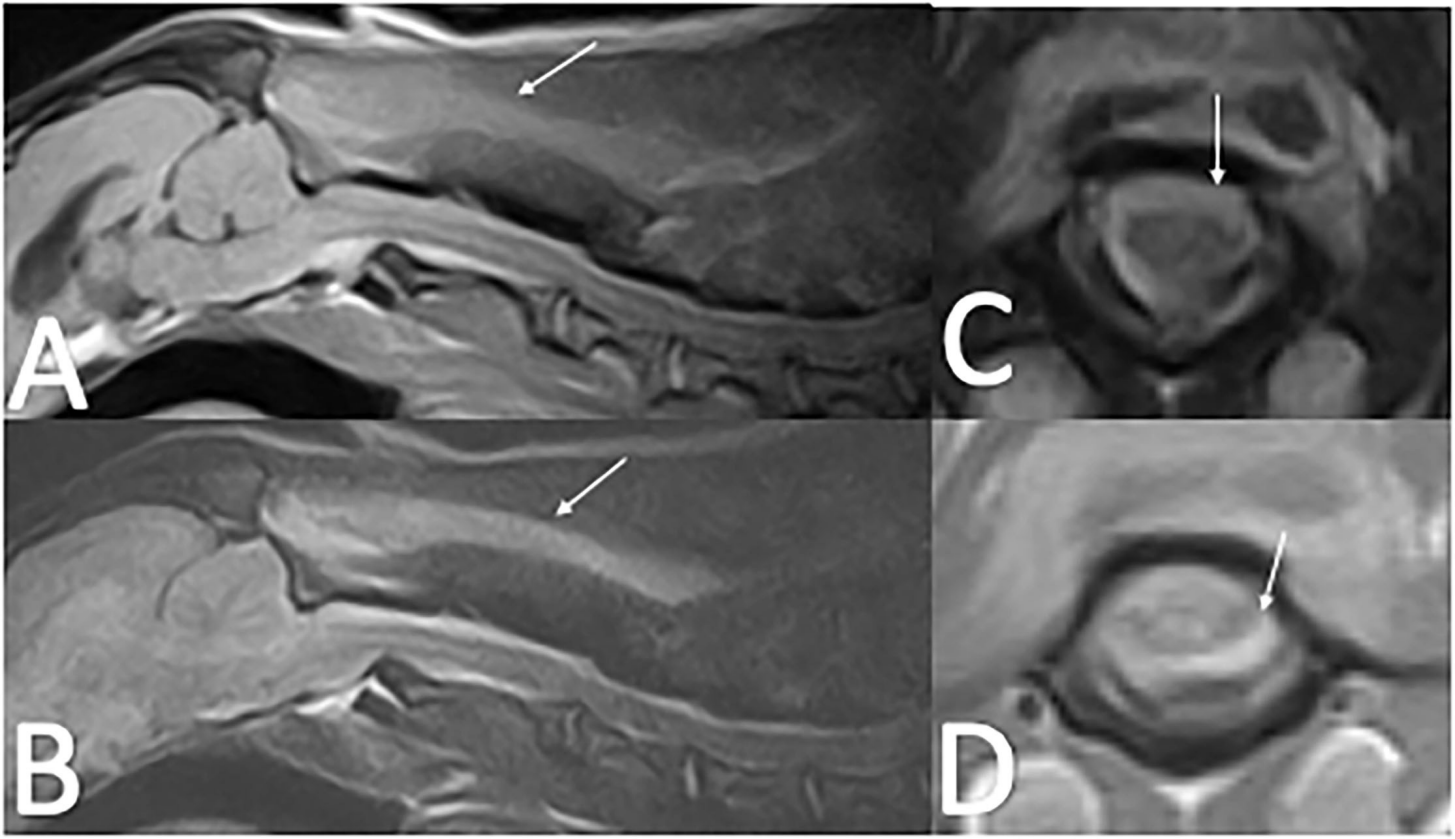
Repeat Cervical MRI of case two following cerebellomedullary cisternal CSF tap. **(A)**—T2W sagittal image of the cervical spine with T2 hyperintensity along the dorsal cervical musculature fascial planes (arrow) with increased T2 hyperintensity dorsal to the spinal cord at C1–C2 compared to Figure 2. **(B)**—T1W sagittal image of the cervical spine with T1 hyperintensity along the dorsal musculature fascial planes (arrow). **(C)**—T2W transverse image at the level of C1 with T2 iso to hyperintense material resulting in spinal cord compression (arrow). **(D)**—T1W transverse image at the level of C1 with T1 iso- to hypointense material resulting in spinal cord compression.

Cervical radiographs performed by the referring veterinarian were normal. A cervical MRI appeared normal on T2W and T1W pre-contrast images. However, there was meningeal T1W gadolinium contrast enhancement extending from C1 to C4. Images were acquired with a 0.25T MRI^2^. Differentials following MRI included meningitis and neoplasia, in particular round cell neoplasia (Figure 2). Cisternal and lumbar CSF samples were collected. Cell counts were not able to be obtained as both samples were consistent with hemorrhage. Lumbar CSF cytology revealed low cellularity with neutrophils (39%) and hemosiderin-laden macrophages (42%), with smaller numbers of lymphocytes (16%), eosinophils (2%), and plasma cells (1%). A large hematoma developed at the site of the lumbar spinal tap. Clotting times revealed a normal PT (13.9 s; reference interval, 11–16 s) and a prolonged aPTT (28 s; reference range, 9.9–12.1 s). During anesthetic recovery following the MRI and spinal tap, the patient became apneic and had to be reintubated and mechanically ventilated. Additional treatment consisted of naloxone 0.014 mg/kg IV once, mannitol 0.5 gram/kg IV over 20 min twice, and dexamethasone-SP 0.07 mg/kg IV once. A cervical MRI was repeated and revealed a large focus of T1W and T2W hyperintensity between fascial planes of the cervical dorsal spinal musculature. There was also extradural material at the level of C1 that was T1W hyperintense and T2W iso- to hyperintense. This was interpreted as hyperacute hemorrhage (Figure 3). During recovery after the second MRI, the dog exhibited multiple cranial nerve deficits and mechanical ventilation was required. The owners elected euthanasia without a necropsy. A hemophilia panel revealed a factor VIII deficiency (3%; reference range, 50–200) consistent with a diagnosis of hemophilia A.

### Case 3

**Figure 4:**
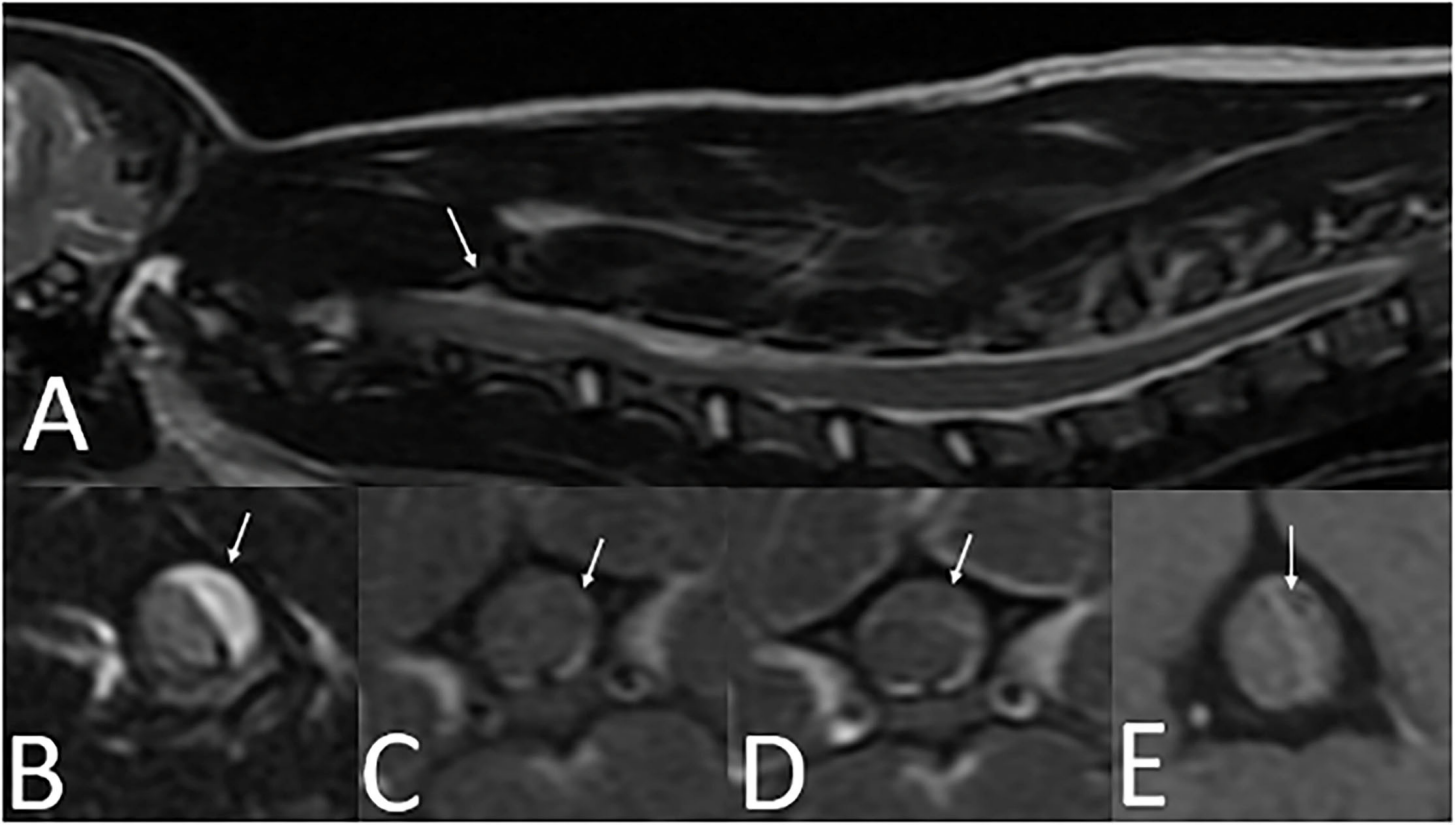
Cervical MRI in a 5-month-old 2.5 kg sexually intact male Yorkshire Terrier with an acute onset of cervical pain (case three). **(A)**—T2W sagittal image of the cervical spine with longitudinal T2 hyperintensity extending from C2-C6 (arrow). **(B)**—T2W transverse image at the level of C4 showing T2 hyperintense extradural material resulting in severe spinal cord compression (arrow). **(C)**—T1W pre-contrast transverse image at the level of C4 showing T1 iso- to hypointense extradural material resulting in severe spinal cord compression (arrow). **(D)**—T1W post-contrast image at the level of C4 showing minimal contrast enhancement along the rim of the extradural material (arrow). **(E)**—T2* image at the level of C4 with heterogenously hyperintense extradural material in the left dorsolateral vertebral canal. A susceptibility artifact is present (arrow).

A 5-month-old 2.5 kg sexually intact male Yorkshire Terrier (Dog 3) was evaluated for an acute onset of cervical pain and a several day history of progressive neurologic deficits. Physical examination revealed ecchymoses on the ventral abdomen which was suspected to be secondary to a previous cystocentesis. On neurologic examination he was non-ambulatory, with severe tetraparesis and cervical hyperesthesia. The neuroanatomic localization was a C1-C5 myelopathy. A CBC and serum biochemistry panel performed with the primary veterinarian were within normal limits. A cervical MRI revealed extradural T2W hyperintense and T1W iso- to hypointense material with minimal rim contrast enhancement that extended from C2–C6 (Figure 4). T2^*^ imaging revealed the presence of mixed hyperintensity and signal void consistent with a susceptibility artifact at the level of C2, C4 and C5. Images were acquired with a 1.5T MRI^3^. Differentials following MRI included meningitis with secondary empyema or hemorrhage, hemorrhage, and neoplasia. Clotting times were performed and revealed a normal PT (7.9 s; reference interval, 7–9.3 s) and a prolonged aPTT (25 s; reference interval, 8.5–9.5 s). Surgical decompression *via* a C2–C6 continuous dorsal laminectomy revealed a severely compressive extradural hematoma which was excised. Due to the concern for a coagulopathy, a single unit of fresh frozen plasma (FFP) and an injection of desmopressin 2 μg/kg were administered intraoperatively. The FFP was continued as a maintenance fluid for 6 h post-operatively.

Following surgery, the dog improved to become ambulatory with a mild tetraparesis. A hemophilia panel revealed a factor VIII deficiency (3%; reference range, 50–200) consistent with a diagnosis of hemophilia A. Two weeks after surgery, his neurologic exam was normal and the ecchymoses on his ventral abdomen had improved. He was discharged with instruction to avoid high impact activities due to the risk of future hemorrhage. No additional follow-up was obtained.

## Discussion

Hemophilia A is an X-linked coagulation disorder that arises as a result of a spontaneous mutation causing a subsequent deficiency in coagulation factor VIII. Prolonged aPTT with a normal PT raises clinical suspicion for hemophilia A, but definitive diagnosis is made by specific factor testing or molecular testing. All three dogs in this case series exhibited these findings of normal PT and prolonged aPTT, which increased the suspicion for hemophilia while specific factor testing was pending. Dogs with hemophilia A are at risk of spontaneous bleeding, which commonly manifests as subcutaneous hematomas, gingival bleeding, hemothorax, hemoabdomen, or excessive hemorrhage following an invasive surgical procedure or trauma. Less commonly, dogs with hemophilia A may experience spontaneous vertebral canal hemorrhage, which may or may not result in spinal cord compression (4). Animals diagnosed with hemophilia A are classified as mild (FVIII 5–25%), moderate (FVIII 2–5%), and severe (FVIII <2%) (17). However, a retrospective study evaluating the outcome of dogs with hemophilia A suggested that the severity of FVIII deficiency may not correlate with the severity of clinical signs or long-term outcome (4). Treatment for hemophilia A in dogs currently consists of replacement of FVIII *via* FFP or cryoprecipitate during spontaneous or anticipated bleeding. In human medicine, recombinant FVIII protein treatment and gene therapy are available (18). Experimental studies in a colony of hemophiliac dogs are investigating gene replacement therapy (19).

All dogs in this case series were presented to a referral hospital for acute onset of paraspinal hyperesthesia or progressive neurologic deficits. The two Dachshunds (cases 1 and 2) are not thought to be related, as they were evaluated 9 years apart, but a familial history was not obtained from either patient. MRI imaging characteristics of hemorrhage can be difficult to characterize in and around the spinal cord. The appearance of hemorrhage in the nervous system depends on the age of the hemorrhage and magnetic field strength and may be more difficult to visualize in low field MRI (20–22). Additionally, there may be species difference between dogs and humans as far as the rate of hemoglobin degradation following hemorrhage into the CNS, but these differences have yet to be characterized fully (15, 16).

MRI findings from case 1 revealed T2W hyperintense, T1W hyperintense, homogenously contrast enhancing extradural material. The differential diagnoses for these imaging findings were late subacute hemorrhage, neoplasia, and meningitis including SRMA or infectious meningitis with secondary hemorrhage or empyema. The presence of a signal void on the T2^*^ supports the presence of hemorrhage. On the second MRI from case 2, there was abnormal T1W and T2W hyperintensity in the cervical musculature and an increase in the volume of T1W and T2W hyperintense material in the vertebral canal in comparison to the first MRI. There were approximately 5 $\frac{1}{2}$ h between the two MRIs, a timeline consistent with hyperacute hemorrhage. No T2^*^ imaging sequence is available for this second MRI in case 2. The timeline for case 1 is very similar to the case presented by Wang-Leandro et al., though in that case the hemorrhage was intradural, while in case 2, it appears to be primarily extradural (9). In case 3, the extradural material was T2W hyperintense, T1W iso- to hypointense, and there was a T2^*^ signal void consistent with a susceptibility artifact. The differential diagnoses for this case were the same as for case 1, other than the hemorrhage was more consistent with acute hemorrhage. Case 3 is similar to the MRI findings in a juvenile keeshond with an extradural hematoma secondary to hemophilia B (6). The MRI used in cases 1 and 3 were 1.5T; in case 2 it was 0.25T. See Table 1 for a summary of the MRI findings in all three cases.

**Table 1:** Timeline and MRI characteristics of cases 1, 2 (second MRI), and 3 with the corresponding estimated age of hemorrhage.

**Case number**	**Reported time from onset of clinical signs to imaging**	**MRI Strength**	**Signal intensity (summary)**	**Approximate age of Hemorrhage based on imaging findings**
Case 1	Approximately 1 month	1.5 T	T1W–iso to hyperintense T2W–heterogenous hyperintense T2–hyperintense with a signal void	Late Subacute
Case 2 (Second MRI)	<6 h	0.25 T	T1W–iso to hypointense T2W– iso to hyperintense T2*–Not performed	Hyperacute
Case 3	Approximately 3 days	1.5 T	T1W–hypointense T2W–hyperintense T2*–hyperintense with a signal void	Acute

Meningitis (steroid responsive meningitis arteritis, infectious, or immune-mediated) was an initial differential diagnosis for all cases presented in this report. Steroid responsive meningitis arteritis (SRMA) typically results in juvenile dogs experiencing severe neck pain, fever, and an inflammatory leukogram. Two of the cases presented also had evidence of extradural compressive material on MRI. Dogs with SRMA can experience secondary extradural or intramedullary vertebral canal hemorrhage (9, 23, 24). This can be difficult to differentiate from hemorrhage secondary to an inherited coagulopathy. Infectious meningitis can lead to extradural spinal cord compression from empyema (25–27). It is possible that a previous hemorrhagic event had occurred, as this has been reported to rarely cause contrast enhancing lesions in human patients (28). It is also reported that intracerebral hemorrhage and the associated hemoglobin breakdown results in inflammation and increased permeability of the blood brain barrier (19, 29). Diagnostics to further differentiate these disease processes following MRI involve additional tests such as specific coagulation factor testing, CSF sampling, and potentially surgery.

CSF sampling was performed in cases 1 and 2. Complications in these cases included subcutaneous hematoma formation at the site of CSF tap in case 1 and extradural hemorrhage resulting in the need for mechanical ventilation and euthanasia in case 2. Hematomyelia has previously been reported in one case after lumbar spinal puncture, but that dog was not reported to have any coagulopathy (8). Case 1 also experienced complications related to its arterial catheter, with subsequent development of a severe anemia necessitating a blood transfusion. Both case 2 and 3 had clinical exam findings suggestive of a coagulopathy, including episcleral hemorrhage and ecchymoses. None of the dogs had a known history of bleeding tendencies, and all three were sexually intact. Other reported exam findings with congenital coagulopathies include subcutaneous hematoma and gingival hemorrhage (4). Often, animals with inherited coagulopathies have a history of excessive hemorrhage during surgery, prolonged bleeding following injection or injury, or intermittent lameness (4). Coagulation testing should be pursued in patients with a compatible history and exam findings for a coagulopathy. Case 3 improved after surgical decompression, indicating that with appropriate supportive care, surgical intervention may be beneficial in dogs with a myelopathy secondary to hemophilia. Cases 1 and 2 in this series had an overall poor outcome and had a moderate FVIII deficiency. While the long-term prognosis of case 3 with a moderate FVIII deficiency is unknown, there was a positive outcome for at least 2 weeks after surgery. This is consistent with a previous report that determined the FVIII concentration may not correlate with clinical severity of spontaneous bleeding (4).

Depending on the geographical area, another important differential diagnosis for hemorrhage into the vertebral canal is *Angiostrongylus vasorum*, which is common in Europe but rare in the United States (30). *A. vasorum* is a nematode that infects dogs as an intermediate host after ingesting snails or slugs. This often results in a secondary coagulopathy and pulmonary hemorrhage, but aberrant migration to the central nervous system has been previously documented (31). MRI findings of the brain and spinal cord in dogs infected with *A. vasorum* revealed lesions that were T2W hyperintense, T1W iso-hypointense, and contained T2^*^ signal voids (32). This was not considered as a likely differential in these three cases, as they were all located in the United States.

Based on the findings from this case series, hemophilia A should be a differential for young, male dogs presenting with an acute onset of paraspinal hyperesthesia and/or progressive neurologic deficits, while caution should be taken when performing CSF sampling or surgery. This is especially true of dogs with other visible evidence of spontaneous hemorrhage such as scleral hemorrhage or ecchymoses, as was noted in cases two and three. The use of MRI and particularly gradient echo imaging may be beneficial to suggest the presence of hemorrhage, though there are few studies carefully characterizing the progression of hemorrhage over time in the spinal cord microenvironment. Due to the specific measurement of coagulation factors often not being readily available, screening coagulation assays for PT and aPTT are highly recommended in these patients. These can quickly be performed prior to CSF sampling and may indicate a coagulopathy in patients with a normal platelet count and buccal mucosal bleeding time.

## Data Availability Statement

The raw data supporting the conclusions of this article will be made available by the authors, without undue reservation.

## Ethics Statement

Ethical review and approval was not required for the animal study because it was conducted following the previously established guidelines of UC Davis, Virginia Tech, and the Virginia-Maryland College of Veterinary Medicine. Written informed consent was obtained from the owners for the participation of their animals in this study.

## Author Contributions

KF, RP, AA, JR, and KV reviewed neuroimaging studies. KF and RP drafted the manuscript. All authors participated in the clinical case management, meet the criteria for authorship, participated in the review, and editing of the manuscript.

## Conflict of Interest

The authors declare that the research was conducted in the absence of any commercial or financial relationships that could be construed as a potential conflict of interest.

## Publisher's Note

All claims expressed in this article are solely those of the authors and do not necessarily represent those of their affiliated organizations, or those of the publisher, the editors and the reviewers. Any product that may be evaluated in this article, or claim that may be made by its manufacturer, is not guaranteed or endorsed by the publisher.

## Footnotes

^1^MRI Philips Intera 1.5T.

^2^MRI Esaote 0.25T.

^3^MRI GE Signa 1.5T.

## Abbreviations

FVIII: Factor VIII
FFP: Fresh frozen plasma
PT: Prothrombin time
aPTT: activated partial thromboplastin time
T1W: T1-weighted MRI sequence
T2W: T2-weighted MRI sequence
T2^*^: T2^*^-weighted gradient echo MRI sequence.
